# Retraction: Optimized Scorpion Polypeptide LMX: A Pest Control Protein Effective against Rice Leaf Folder

**DOI:** 10.1371/journal.pone.0312334

**Published:** 2024-10-14

**Authors:** The PLOS ONE Editors

Following the publication of this article [1], concerns were raised regarding results in multiple figures. Specifically,

- In Figures 3A and 7A, there are 7 pairs of images affecting 14 panels that appear to present the same or overlapping data. In most cases, the similar images are used to represent results for different experimental conditions.
- 14 panels in Figures 3A appear similar to results reported for different experiments in Figures 1 and 4 of [2], which was published after this article [1].
- In Figure 1B, there is a vertical discontinuity in the background between lanes labeled 4 and 5 suggestive of splicing.
- In Figure 4A, the LMX 5.0μg right image appears similar to the LMX 10μg left image.
- Lanes P and 3–7 in Figure 6E of [1] appear similar to lanes P and 4–8 in Fig 2E of [2].

Image files provided in response to these issues were insufficiently labelled to identify which experimental groups they represented, and did not resolve the above concerns.

The *PLOS ONE* Editors retract this article due to concerns about the reliability of the reported results.

XT agreed with the retraction. YZ and SL either did not respond directly or could not be reached.
